# Inclusion of Fermented Foods in Food Guides around the World

**DOI:** 10.3390/nu7010390

**Published:** 2015-01-08

**Authors:** Stephanie N. Chilton, Jeremy P. Burton, Gregor Reid

**Affiliations:** 1 Canadian Centre for Human Microbiome and Probiotic Research, Lawson Health Research Institute, 268 Grosvenor St., N6A 4V2, London, ON, N6A 4V2, Canada; E-Mails: schilto@uwo.ca (S.N.C.); jeremy.burton@lawsonresearch.com (J.P.B.); 2 Department of Physiology and Pharmacology, University of Western Ontario, London, ON, N6A 3K7, Canada; 3 Department of Microbiology and Immunology, University of Western Ontario, London, ON, N6A 3K7, Canada; 4 Department of Surgery, University of Western Ontario, London, ON, N6A 3K7, Canada; 5 Division of Urology, University of Western Ontario, London, ON, N6A 3K7, Canada

**Keywords:** food guides, world, fermented foods, fermentation, benefits, probiotics

## Abstract

Fermented foods have been a well-established part of the human diet for thousands of years, without much of an appreciation for, or an understanding of, their underlying microbial functionality, until recently. The use of many organisms derived from these foods, and their applications in probiotics, have further illustrated their impact on gastrointestinal wellbeing and diseases affecting other sites in the body. However, despite the many benefits of fermented foods, their recommended consumption has not been widely translated to global inclusion in food guides. Here, we present the case for such inclusion, and challenge health authorities around the world to consider advocating for the many benefits of these foods.

## 1. Introduction

Recommendations for the consumption of certain nutritious foods date back to the Hippocratic Corpus of Ancient Greece. More recently, the United States Department of Agriculture first created nutrition guidelines in 1894 which advocated variety, proportionality and moderation, calorie measuring, nutrient-rich foods and consumption of less fat, sugar and starch [1]. Canada’s first Food Guide was introduced in July 1942, to provide guidance to Canadians on proper nutrition during a period when wartime rationing was common [2].

While such guidelines result from consultation with knowledge providers, they need not reflect traditions practiced by populations nor do they appreciate the benefits of foods consumed by generations of ethnic groups. Foods that are prepared by fermentation (the slow decomposition process of organic substances induced by microorganisms, or by complex proteinaceous substances (enzymes) of plant or animal origin [3]), occurs due to biochemical changes brought about by the anaerobic or partially anaerobic oxidation of carbohydrates. This process has long been shown to help retain shelf-life and prevent food spoilage. The absence of fermented foods from some food guides, as will be discussed later, should not be interpreted as suggesting these foods are not beneficial. Rather, they may not have had a history of use in a particular country, and may be made at home instead of being purchased from a commercial enterprise. The aim of the present article is to examine the history of fermented foods, their health benefits and the basis for why they are, or should be, included in the food guides of different countries across the continents. Such a review with evidence of the effectiveness of fermented foods, is one of the means that regulatory agencies, such as Health Canada, use to evaluate whether or not certain foods are worthy of inclusion in a revised food guide.

## 2. Fermented Foods

What exactly are fermented foods? Fermentation is a process that has been used by humans for thousands of years, with major roles in food preservation and alcohol production. Fermentation is primarily an anaerobic process converting sugars, such as glucose, to other compounds like alcohol, while producing energy for the microorganism or cell. Bacteria and yeast are microorganisms with the enzymatic capacity for fermentation, specifically, lactic acid fermentation in the former and ethanol fermentation in the latter. Many different products around the world are a result of fermentation, either occurring naturally or through addition of a starter culture. Different bacterial and yeast species are present in each case, which contribute to the unique flavors and textures present in fermented foods (Table 1). These bacteria and yeasts are referred to as “probiotic” when they adhere to the following World Health Organization (WHO) definition: “live microorganisms which, when administered in adequate amounts, confer a health benefit on the host” [4].

During lactic acid fermentation, the pyruvate molecules from glycolysis are converted into lactate. Lactic acid bacteria (LAB) consist of homo and hetero-lactic acid organisms, and are a broad category of bacteria, including *Lactobacillus*, *Streptococcus*, *Enterococcus*, *Lactococcus* and *Bifidobacterium* [5], with the ability to produce lactate primarily from sugars. They are among the most commercially used bacteria today [6,7], contributing to yogurt, sauerkraut, kimchi [8] and kefir production [9], the pickling of vegetables, curing of fish, and many other traditional dishes around the world [10,11].

In comparison, ethanol fermentation produces carbon dioxide and ethanol from pyruvate molecules, mainly through the actions of various yeasts. *Saccharomyces* *cerevisiae* is used in bread making, helping the dough rise through the production of carbon dioxide. A separate strain of *S. cerevisiae* is also used in alcohol production, including beer and wine, in combination with other yeast species [12].

**Table 1:** Examples of fermented foods and countries in which they are believed to originate and remain particularly popular.

Fermented Food and Main Constituents	Country
Yogurt—milk, *L. bulgaricus*, *S. thermophilus*	Greece, Turkey
Kefir—milk, kefir grains, *Saccharomyces cerevisiae* and *L. plantarum*	Russia
Sauerkraut—green cabbage, *L. plantarum*	Germany
Kimchi—cabbage, *Leuconostoc mesenteroides*	South Korea
Cortido—cabbage, onions, carrots	El Salvador
Sourdough—flour, water, *L. reuteri*, *Saccharomyces cerevisiae*	Egypt
Kvass—beverage from black or rye bread, *Lactobacillus*	Russia
Kombucha—black, green, white, pekoe, oolong, or darjeeling tea, water, sugar, *Gluconacetobacter* and *Zygosaccharomyces*	Russia and China
Pulque—beverage from agave plant sap, *Zymomonas mobilis*	Mexico
Kaffir beer—beverage from kaffir maize, *Lactobacillus* sp.	South Africa
Ogi—cereal, *Lactobacillus* sp., *Saccharomyces* sp., *Candida* sp.	Africa
Igunaq—fermented walrus	Canada
Miso—soybeans, *Aspergillus oryzae*, Zygosaccharomyces, *Pediococcus* sp.	Japan
Tepa—Stinkhead fermented fish	USA
Dosa—fermented rice batter and lentils, *L. plantarum*	India
Cheddar and stilton cheeses—*Penicillium roqueforti*, *Yarrowia lipolytica*, *Debaryomyces hansenii*, *Trichosporon ovoides*	United Kingdom
Surströmming—fermented herring, brine, *Haloanaerobium praevalens*, *Haloanaerobium alcaliphilum*	Sweden
Crème fraîche—soured dessert cream, *L. cremoris*, *L. lactis*	France
Fermented sausage—Lactobacillus, Pediococcus, or Micrococcus	Greece and Italy
Wine—various organisms particularly *Saccharomyces cerevisiae*	Georgia

## 3. Examples of Fermented Foods from around the World

The ability to create tasty food using microbes reflects human culinary innovation at its best. The use of microbial fermenters has been instrumental in making a large range of foods, popular around the world. Examples of these are given in Table 1, illustrating diversity and opportunism by the originators of the food formulae.

These traditional foods have been consumed in some cases for thousands of years, with recipes being passed down through generations, as well-documented elsewhere [13]. Initially, many foods underwent fermentation naturally, but today, a number of them are made with the addition of a starter culture and the process has become automated and more reproducible and reliable. There are clearly types of fermented foods consumed across countries and continents, such as sauerkraut, kimchi and cortido, all products of fermented cabbage. Likewise, some foods remain quite limited in the scope of who consumes them.

A trend in the past 20 or so years has been in the globalization of foods, aided by shipping and airline delivery, and a desire by consumers to gain access to products. Thus, in the depths of winter in Canada, consumers can still purchase “fresh” fruit and vegetables from countries in the southern hemisphere. However, for the most part, global distribution is not required for fermented foods. Instead, they tend to be made locally with outside temperature not being an issue. Often, immigrants will introduce these foods for their own use, then their popularity grows and consumption becomes widespread. The net result is that fermented foods are widely consumed across the globe (Table 2) [14,15].

**Table 2:** Widely consumed fermented foods, the country they are consumed in, and the average amount of consumption per person annually.

Food	Country	Average Annual Consumption (per Person)
Beer	Germany	106 L
Cheese	UK	10 kg
Kimchi	Korea	22 kg
Miso	Japan	7 kg
Soy Sauce	Japan	10 L
Tempeh	Indonesia	18 kg
Wine	Italy, Portugal	90 L
	Argentina	70 L
	Finland	40 L
Yogurt	Netherlands	25 L

## 4. Nutritional Guides from around the World

**Figure 1:**
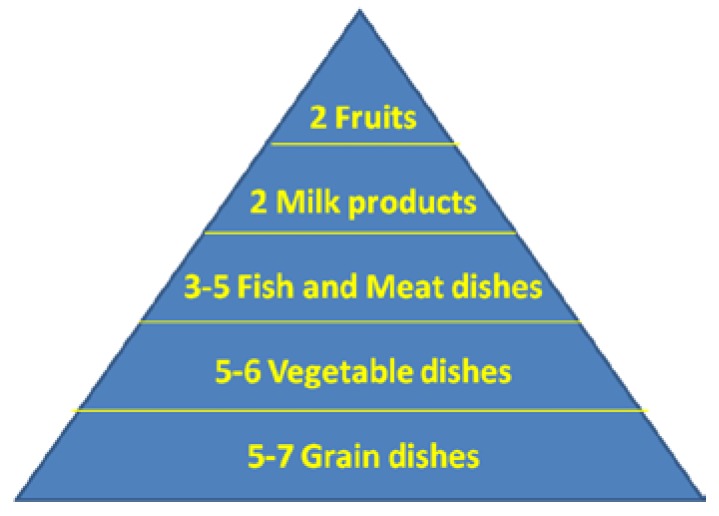
Breakdown of the food groups in the Japanese Food Guide pyramid, with portions per day.

**Figure 2:**
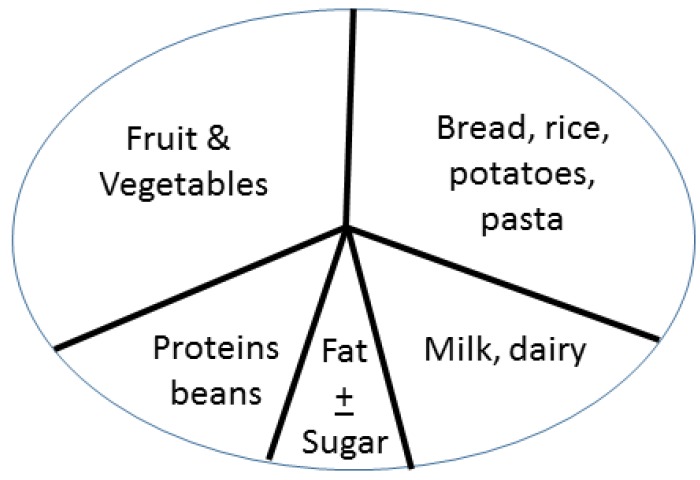
The pie or plate design illustrates the British Food Chart with emphasis on carbohydrates, fruit and vegetables, and no category of fermented food.

Nutritional guides around the world come in many different formats, illustrated as pyramids, pie charts, text and tables, yet they are similar in terms of content. Japan, like most countries, states the importance of every food group taken daily in moderation in order to achieve a well-balanced diet, but it emphasizes more carbohydrates than proteins and does not specifically highlight fermented foods as a category (Figure 1). In Canada and the USA, food guides have yogurt and kefir as recommended items listed under the dairy products section, but there is no emphasis on them being fermented foods, nor is there inclusion of fermented foods as a healthy category. The United Kingdom presents their Food Guide as a plate, with emphasis on carbohydrates, fruits and vegetables (Figure 2). The Swedish model for healthy eating, also in the form of a plate, has no section allotted to dairy products or any fermented foods. They only stress the importance of consuming foods that are low in fat and high in fiber at every meal. 

Given the history of fermented foods in Asia, it is surprising that Japan and China, for example, do not recommend this as a category in their Food Guides. China’s “food pagoda” stresses the importance of dietary balance and places crude wheat, rice, corn and sorghum cereals as “Level 1” for energy sources. The Chinese Nutrition Society (CNS) does suggest the use of yogurt for those who do not tolerate milk [16]. The one exception in Asia is India, whose Guide explicitly encourages the consumption of fermented foods. The National Institute of Nutrition’s 2010 “Dietary Guidelines for Indians” document suggests specifically to pregnant women that they should: “eat more whole grains, sprouted grams and fermented foods” [17]. The document also describes the enhanced digestibility of fermented foods and increased nutritional value, through greater production of vitamins B and C. Again, fermented foods are encouraged later in the document when discussing various methods of food preparation. In Japan, probiotics are listed in “Food for specified health uses” (FOSHU), allowing labelling of health-promoting functions.

With few exceptions, fermented foods are generally absent as a recommended category of food for daily intake, in Food Guides. We believe this reflects a failure to appreciate the benefits resulting from the process of fermentation, which have been supported by numerous studies. When individual fermented foods such as yogurt are included, it is because of their nutritional value, such as high calcium levels.

## 5. Benefits of Fermented Foods

### 5.1. Benefits of Fermented Dairy Products

Fermented foods and the microorganisms that contribute to the fermentation process have been associated with many beneficial effects on human health. Recent large cohort studies in the Netherlands and Sweden have examined the effects of regular consumption of fermented dairy products on the risk of bladder cancer [18] and cardiovascular disease [19]. Dairy products were divided according to those fermented and those that were not. In both studies, only fermented milk products were significantly associated with decreasing disease prevalence. In another large study on Danish participants, the effects of dairy products on periodontitis were examined [20]. It was reported that calcium intake specifically associated with fermented foods was inversely and significantly correlated with periodontitis, while calcium from other dairy foods was not. These findings emphasize the need to differentiate the types of dairy products, fermented and non-fermented, with regards to their health benefits, instead of promoting all dairy products, as is the case with many food guides.

The basis for fermented dairy products conferring health effects, in addition to the nutritional value of non-fermented milk, is multi-fold. The proteolysis that occurs in fermenting milk results in a higher content of peptides and free amino acids, especially cystine, histidine, and asparagine [21], which play various roles in health, and produce a more digestible food than milk *per se*. The breakdown of lactose concentration by the bacteria containing β-galactosidase, not only in the fermentation process but also in the stomach when the bacteria die and release this enzyme, then allows many lactose-intolerant individuals to consume the milk product. Lactose-free products are available for particularly sensitive individuals. Although the level of thermostable vitamins, niacin and pantothenic acid, are not destroyed by milk pasteurization that occurs prior to fermentation, some LAB can resynthesize folates, which are destroyed by the heat and have been shown to confer many health benefits [22,23]. Although there is some evidence that fermented foods alleviate constipation [24], studies using probiotic *B. lactis* DN-173 010 [25] or *L. casei* Shirota fermented milk [26] were no better than control products in showing a difference in constipation severity and stool frequency over a three or four-week period. 

The two main health effects from fermented dairy consumption are immune and metabolic, especially with the addition of probiotic organisms. Fermented milk supplemented with probiotics can improve intestinal health, humoral and cell-mediated immunity [27], and salivary and fecal antibodies [28,29]. Some evidence suggests that this can lead to reduced incidence, or duration, of respiratory infections [30,31,32] presumably because the priming at the intestinal mucosal level impacts the lung’s immune response. This is just one example of distant site health effects of probiotic fermented foods, but many others occur, including improvements to vaginal [33], bladder [34], bone [35], liver [36,37], body mass and blood pressure indices [38] and skin health [39]. Interestingly, a study of children from a low socioeconomic area of Argentina had no elevation of antibodies irrespective of vaccinations, suggesting that their system was already primed by exposure to pathogens [40].

The modulation of immune parameters is particularly challenging in two extreme conditions: inflammatory bowel disease (IBD) and human immunodeficiency virus (HIV) infection. The safe use of fermented foods in these types of extreme cases is important if they are to be recommended as part of a national food guide. As IBD is a Th1 immune response, treatment requires administration of anti-inflammatory therapy, such as antibodies to TNFα, or probiotic yogurt, which increases Treg cells [41]. Overall, the data are limited for remediation of IBD using probiotics, but some studies are supportive [42]. The Th2 immune response in HIV-infected patients associated with depletion of CD4+ and dendritic cells can lead to compromised epithelial repair mechanisms and enhanced epithelial permeability. Probiotic fermented foods can help maintain epithelial layer integrity [43,44], and reduce the loss of CD4+ cells in HIV patients [45,46]. In the latter group, maintenance of gut barrier function also helps reduce bacterial translocation that can cause serious infectious complications to the immune-suppressed host. 

### 5.2. Benefits of Fermented Foods in Vulnerable Populations

Food guides are designed for the general public, not hospitalized patients. Nevertheless, the studies showing benefits of probiotic food to aid recovery from organ transplantation and abdominal surgery [47,48,49,50] further demonstrate safety and effectiveness. An important safety study of infants randomly assigned to receive probiotics or placebo for a total of five months, starting two months prior to vaccination, showed no adverse interference with the immune response to mumps, measles, rubella and varicella vaccine [51].

The high prevalence of allergies affecting skin, gut and respiratory tract, have led to probiotics being tested in humans as a means of prevention or treatment. In a nested unmatched case-control study, 237 infants were given probiotics prenatally and at 6 months of age. By age 2, the risk of eczema, food allergy, asthma, and rhinitis was assessed [52]. In infants with high fecal IgA concentration at 6 months, the risk of having any allergic disease tended to reduce (odds ratio (OR): 0.52), as did the risk for any IgE-associated (atopic) disease (OR: 0.49). High fecal calprotectin at the age of 6 months was associated with lower risk for IgE-associated diseases (OR: 0.49). This study showed the broad potential of probiotic food against allergy. In a study of almost 200 infants aged 4 to 13 months, use of probiotic cereal showed signs of preventing early manifestation of allergy, and the higher Th1/Th2 ratio suggested an effect on the T-cell-mediated immune response [53]. It is not unexpected that foods are less able to treat conditions like atopic dermatitis [54], but their demonstrated safety in such infants is reassuring. Likewise, in adults prone to seasonal grass and ragweed allergies, a pilot study showed some markers of success and no adverse responses [55].

The increasing diabetes rates amongst pregnant women and children have led to consideration of probiotic intervention. In a study undertaken in Iran, daily consumption of probiotic yogurt for 9 weeks maintained serum insulin levels, potentially preventing pregnant women from developing insulin resistance [56]. Preservation of insulin sensitivity [57] and controlling glycemic index are very important for patients at risk of, or suffering from, diabetes [58,59]. A recent study with an impressive nested case-cohort and a random sub-cohort of 4000 subjects followed-up for 11 years showed that greater low-fat fermented dairy product intake was associated with a decreased risk of type 2 diabetes development [60]. Thus, with pre-diabetes and type II diabetes affecting several million Canadians, the inclusion of fermented foods in their diet could be significant.

Considering that arthritis is even more prevalent, affecting almost five million Canadians, and the influence that the microbiome has on arthritis [61], it is worth investigating whether fermented foods and probiotics could alleviate pain, swelling and discomfort. Reactive arthritis is known to be triggered in some patients by Gram-negative gastrointestinal infection. A mouse study showed that consumption of probiotic fermented milk prevented *Salmonella*-induced synovitis by altering the intestinal milieu necessary for differentiation of cells involved in the generation of joint inflammation [62]. A small human study on rheumatoid arthritis, in patients with at least four swollen and four tender joints and stable medications with no steroids for at least one month prior to and during the study, showed a significant improvement in the Health Assessment Questionnaire score after three months of probiotic treatment [63]. This illustrates that probiotics recommended in a food guide, will not cause harm even in subjects who might consume such products while suffering from common arthritic disease.

A large portion of populations in developed countries take a range of prescription medications, most of which confer adverse effects of one sort or another. Indeed, large numbers of patients admitted to emergency clinics do so following drug complications, with agents used to manage cardiovascular disease being the main culprits [64,65]. As fermented foods are known to provide benefits to managing some cardiovascular risk factors [66] and potentially even improve recovery post-infarction [67], the reduction by probiotic food intake of drug side effects [68,69], especially antibiotics [70,71], further suggests benefits, and no harm, if taken by a general population, some of whom are receiving drug therapy.

The intake of LAB can help to reduce the load of pathogens, even in the nasal cavity [72]. This may be an unusual attribute with respect to a food, but since *Staphylococcus aureus*, *Streptococcus* *pneumoniae*, and β-hemolytic streptococci are major causes of disease, the ability of probiotic yogurt to deplete them should be seen as a positive outcome, and therefore something aligned with the expectations from any recommended food type.

### 5.3. Other Fermented Foods and Benefits

Not every consumer eats dairy products, and not every fermented food requires use of milk. Kimchi, fermented vegetables, is a popular side dish originating in Korea that has been associated with numerous health benefits, including prevention of cancer and obesity, reduction in cholesterol levels and immune system promotion [73]. A study that looked at the preventative effects of kimchi, fresh and fermented, in pre-diabetic individuals showed promising results. Insulin resistance decreased while insulin sensitivity increased, and overall, glucose tolerance improved by 33%, compared to only a 9.5% increase in those receiving fresh non-fermented control [74].

The long history and wide diversity of fermented foods across African countries attests to the benefits they have accrued over many generations [75]. Studies have attributed benefits to include prevention of diarrhea and constipation [76]. Researchers there, and in other developing countries with a history of fermented food production, have been examining the properties of strains in their products. A *Lactobacillus plantarum* strain isolated from the common Indian fermented food Dosa, has been shown to inhibit the growth of a range of food-borne pathogens [77].

### 5.4. Adverse Effects of Fermented Foods

In parts of Asia, fermented fish sauce is widely consumed. A study performed in the Chaoshan area of China, showed an increased risk of squamous cell carcinoma of the esophagus in habitual consumers of fermented fish sauce [75]. Another Chinese study showed that *N*-nitroso compounds and genotoxins present before and after nitrosation, appear to be responsible for the cancer risk [76]. An Egyptian study also found high levels of histamine in fermented fish [77].

## 6. Conclusions and Recommendations

The expansive use of, and benefits gained from, fermented foods supports their greater inclusion in Food Guides around the world. They have long been a part of the human diet, and with further supplementation of probiotic microbes in some cases, they offer nutritional and health attributes worthy of recommendation of regular consumption. It is hoped that this review contributes to policy changes and increases the inclusion of fermented foods when Food Guides are next revised. This might, for now, exclude fermented fish consumed in parts of Asia. It would be a great detriment to human health if fermented food use were to decline, as has been noted in parts of Africa, through lack of generational transfer of knowledge, poor availability and affordability of probiotics [78,79,80,81], and introduction of food and drink products high in certain sugars [82,83,84].
